# Modeling of systematic errors in stereo-digital image correlation due to camera self-heating

**DOI:** 10.1038/s41598-019-43019-7

**Published:** 2019-04-25

**Authors:** Liping Yu, Gilles Lubineau

**Affiliations:** King Abdullah University of Science and Technology (KAUST), Physical Sciences and Engineering Division (PSE), COHMAS Laboratory, Thuwal, 23955-6900 Saudi Arabia

**Keywords:** Optical sensors, Mechanical engineering

## Abstract

Errors in strain measurements in stereo-digital image correlation (stereo-DIC) caused by camera self-heating have been experimentally observed in previous research, and have been shown to widely vary from one system configuration to another. Such “thermal errors” are sometimes so large that they strongly compromise the accuracy of the measurements. Despite correcting such errors is crucial when aiming at high-accuracy measurements, the mechanism of the thermal error generation and how it relates to the camera parameters in stereo-DIC are still not clear. In this paper, we first explain in detail how self-heating can introduce large artifacts in the strains measured by stereo-DIC. Using a simplified stereovision model, we provide the main equations that describe the theoretical errors in 3D coordinate reconstruction and 3D deformation measurement. Through several sets of simulations, the effect of camera self-heating on the 3D coordinate, displacement and strain measurements, and the effect of camera parameters on the thermal errors in stereo-DIC were explicitly presented based on the derived theoretical formulas. Finally, several real self-heating tests using a smartphone-based single-camera stereo-DIC system confirm the correctness of theoretical analyses and simulation results.

## Introduction

Stereo-digital image correlation (stereo-DIC) is a well-assessed non-contact optical technique capable of performing 3D shape and deformation measurements across a wide range of length and time scales^1–3^. Compared with two-dimensional digital image correlation (2D-DIC), stereo-DIC can realize 3D shape and deformation measurements of both planar and curved objects without being affected by out-of-plane translations and rotations on the specimen surface, thus offering wider applicability and higher measurement accuracy. With the continuous improvements in reconstruction algorithm and system hardware, stereo-DIC has prone to be a promising tool in scientific researches and engineering applications, such as material properties characterization of various materials^4,5^, multi-scale deformation determination^6,7^, dynamic deformation tracking^8–10^ and 3D deformation measurement in harsh environment^11,12^.

During the implementation of stereo-DIC, digital image pairs of a calibration target with changing positions and/or orientations and a test object at different loading conditions (times or states) are first recorded by two synchronized cameras or a single camera with auxiliary optical devices^13^. The image pairs of the calibration target are processed to determine the camera parameters (including intrinsic and extrinsic parameters), and the correspondence of the projections in the image pairs of the test object are established using correlation algorithms. Together with the calibrated camera parameters, 3D coordinates of measurement points can be reconstructed based on the determined image coordinates of their projections in the two cameras and the triangulation principle. Usually, the calibrated camera parameters for establishing the world coordinate system are assumed constant during the test, which means all the components inside the imaging system will not change in position or orientation. However, in real situations, the camera parameters do not meet this assumption. For a regular stereo-DIC system, the integrated circuit boards of two cameras will generate heat after being switched on, and the heat then transfers to the other components of the cameras, resulting in slight thermal deformation of the mechanical components of the two cameras. This effect is usually known as camera self-heating or camera warm-up effect. As a result, the structural configurations (e.g., the positions and orientations of the camera lens and sensor) inside the cameras will change accordingly due to slight thermal deformation. In other words, the originally calibrated camera parameters and the established world coordinate system of the stereo-DIC systems are changed by the temperature variations. Unavoidably, these slight changes will cause measuring errors in the measured results. To realize high-accuracy 3D shape and deformation measurements, the errors in stereo-DIC caused by the camera self-heating should be carefully studied and then eliminated when possible.

In literature, the camera self-heating phenomenon was observed in the early 1990s by several researchers in machine vision field^14,15^. They found that the image coordinate would drift to some tenth of a pixel during the first hour after camera start-up. To explain the reasons for the observed image coordinate drift, Handel^16^ and Podbreznik and Potocnik^17,18^ both established mathematical models based on pinhole model for modeling the effect of camera self-heating. Their models assume that temperature will not affect the intrinsic camera parameters and only translation of the camera needs to be considered. In fact, the temperature variation will cause thermal expansions of all the components in the camera, and thus their assumptions may be not accurate in some cases. To address this limitation, Yu *et al*.^19^ proposed a more rigorous model to describe the relationship between variations in the camera parameters and drift in the image coordinates, which consider the changes of the intrinsic parameters. Recently, the camera self-heating phenomenon was found in 2D-DIC measurement with a maximum strain error measured as tens to hundreds of microstrains for different cameras^20–24^. Later, Ma *et al*.^22^ experimentally studied the deformation of the camera components and found the difference in the out-of-plane translations of the camera lens and camera sensor. The theoretical displacement and strain errors in 2D-DIC due to camera self-heating were then derived and fully understood according to these observations. More recently, similar temperature-dependent thermal errors were found in stereo-DIC measurement by Pan *et al*.^25^. Our recent research further indicated that the thermal errors would significantly increase to tens of thousands microstrains for a single-camera stereo-DIC system with small baseline distance and short focal length^26^. However, compared with 2D-DIC using one camera, the mechanism of the thermal error generation in stereo-DIC is more complicated as the structural changes of the two cameras should be considered in the triangulation. To understand the thermal errors in stereo-DIC and explain the difference of the errors in different system configurations, a comprehensive theoretical analysis of the errors in stereo-DIC due to camera self-heating is therefore necessary.

In this work, the mechanism of the thermal error generation in stereo-DIC is first clearly explained through two different models. Then, a comprehensive theoretical analysis is performed to quantitatively investigate the 3D reconstruction and deformation errors in stereo-DIC due to camera self-heating based on a simplified stereovision model. The effect of camera self-heating on the full-field 3D coordinate, displacement and strain measurements and the effect of camera parameters on the thermal errors are presented by conducting a series of simulation tests. Finally, real self-heating tests using a smartphone-based single-camera stereo-DIC system were performed on a flat plate to verify the correctness of the theoretical analysis and simulation results.

## Basic Principles of Stereo-DIC Technique

**Figure 1:**
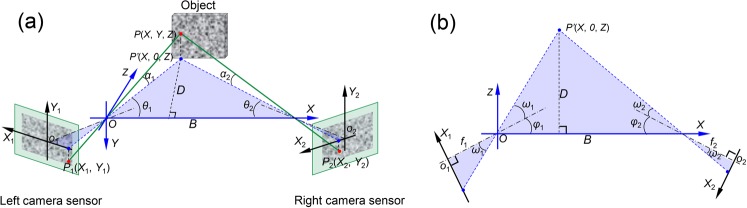
(**a**) Schematic diagram of the imaging model of a stereo-DIC system and (**b**) the geometrical relationships in *XOZ* plane (*O*-*XYZ* is the world coordinate system of the stereo system defined at the optical center of the left camera).

To facilitate the analysis of the thermal errors in stereo-DIC due to camera self-heating, a simplified imaging model based on the pinhole model is adopted here. In this simplified model, the lens distortion and skew of the sensor plane are neglected, and the location of the principal point is assumed to be precisely in the center of the camera sensor. Figure 1 shows the schematic diagram of the imaging model of a stereo-DIC system and its geometrical relationships in the projection plane. As shown in the figure, assuming a physical point *P*(*X*, *Y*, *Z*) on the object surface has been identified on the left and right sensor planes as *P*_1_(*X*_1_, *Y*_1_) and *P*_2_(*X*_2_, *Y*_2_), then the three-dimensional coordinates of this point in the world coordinate system (*O*-*XYZ*, defined at the optical center of left camera) can be derived through geometric relationship as^27,28^:

1 $$\begin{array}{ccc}X & = & \frac{B}{\cot \,{\theta }_{1}+\,\cot \,{\theta }_{2}}\,\cot \,{\theta }_{1}\\ Y & = & \frac{B}{\cot \,{\theta }_{1}+\,\cot \,{\theta }_{2}}\frac{\tan \,{\alpha }_{1}}{\sin \,{\theta }_{1}}\\ Z & = & \frac{B}{\cot \,{\theta }_{1}+\,\cot \,{\theta }_{2}}\end{array}$$

where *θ*_1_ = *ω*_1_ + *φ*_1_, *θ*_2_ = *ω*_2_ + *φ*_2_, *f*_1_ and *f*_2_ are the focal lengths of the two cameras, *φ*_1_ and *φ*_2_ are the angles between the optical axes of the cameras and the baseline, *ω*_1_ and *ω*_2_ are the angles between the horizontal projection of the light rays *PP*_1_ and *PP*_2_ and the optical axes, and *α*_1_ and *α*_2_ are the corresponding vertical projection angles, and *D* is the distance between the projection point *P*’ and the baseline (*D* is equal to *Z* in this world coordinate system). Note that *ω*_1_, *ω*_2_, *α*_1_ and *α*_2_ are not independent variables but can be represented by other structural parameters and coordinates in sensor plane as tan*ω*_1_ = *X*_1_/*f*_1_, tan*ω*_2_ = *X*_2_/*f*_2_, tan*α*_1_ = *Y*_1_·cos*ω*_1_/*f*_1_ and tan*α*_2_ = *Y*_2_·cos*ω*_2_/*f*_2_, respectively. With these parameters and geometric relationships, *θ*_1_, *θ*_2_ and *α*_1_can be expressed as,

2 $$\begin{array}{rcl}{\theta }_{1} & = & \arctan \frac{{X}_{1}}{{f}_{1}}+{\phi }_{1}\\ {\theta }_{2} & = & \arctan \frac{{X}_{2}}{{f}_{2}}+{\phi }_{2}\\ {\alpha }_{1} & = & \arctan (\frac{{Y}_{1}}{{f}_{1}}\,\cos (\arctan \frac{{X}_{1}}{{f}_{1}}))\end{array}$$

Once the camera parameters (intrinsic parameters *f*_1_ and *f*_2_, and extrinsic parameters *φ*_1_, *φ*_2_, and *B*, calibrated using a calibration target) and the coordinates of the two projection points (*P*_1_(*X*_1_, *Y*_1_) and *P*_2_(*X*_2_, *Y*_2_), tracked through stereo-matching process) are determined, the 3D coordinates of point *P* in the world coordinate system (*O*-*XYZ*) can be calculated according to Eq. (1). After repeating this procedure for all the calculation points evenly spaced in the pre-defined region of interest (ROI), the 3D shape of the test object at initial state can be retrieved. Then, the 3D shapes of the test object at deformed states are determined with the same camera parameters and the changed coordinates of the two projection points. By subtracting the reconstructed 3D coordinates of the deformed states from those of the initial state, 3D full-field displacement fields on the specimen surface of different deformed states are retrieved. Finally, the full-field strain maps of the deformed states are calculated by differentiating the displacement fields using a pointwise least square strain estimation approach.

## Theoretical Analysis of The Errors in Stereo-Digital Image Correlation Due to Camera Self-Heating

### The mechanism of the thermal error generation in stereo-DIC

As introduced above, the 3D coordinates of a point on the test specimen are determined by the calibrated camera parameters and coordinates of the two projection points in the left and right images according to the triangulation principle (Eq. (1)). Usually, the camera parameters are calibrated before or after test and regarded as constants for all the image pairs recorded in a test, while the image coordinates of the two projection points vary from each physical point on the object surface to another. Ideally, the camera parameters and image coordinates of the two projection points should be constant when the cameras and the test sample are all kept stable. However, in the real tests, the structural configurations (e.g., the positions and orientations of the camera lens and sensor) inside the cameras will be changed due to slight thermal deformation. These temperature-dependent changes further will lead to slight alterations in positions (i.e., image coordinates) of the two projection points. With the temperature-dependent image coordinates of the two projection points and constant camera parameters, the reconstructed 3D coordinates will change with the temperature according to the triangulation principle.

**Figure 2:**
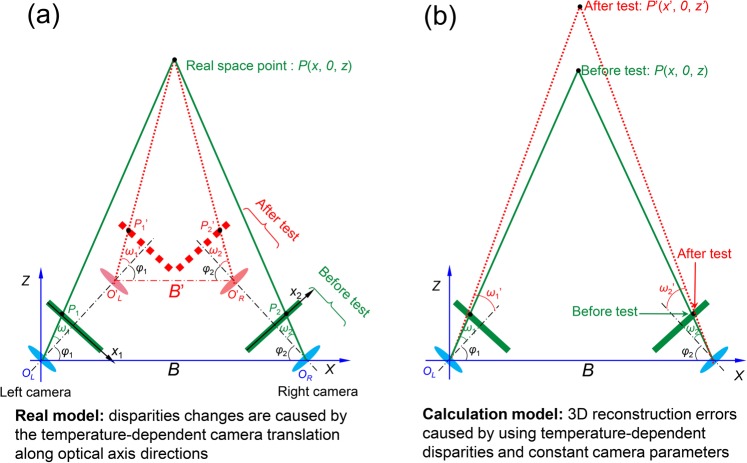
(**a**) A real model to explain how the disparities of the two projection points change during the test, (**b**) A calculation model to explain how the reconstruction errors are introduced during the calculation process. To facilitate the analysis, the sensor plane was symmetrically placed before the imaging lens.

To better understand the errors in the stereo-DIC system due to the camera self-heating, here we give two models, including a real model and a calculation model, to explain how the image coordinates and disparities of the two projection points change during the real test and how the reconstruction errors are introduced during the calculation process, respectively. As shown in Fig. 2(a), assuming the two cameras have temperature-introduced translations along the optical axis directions, the two projection points of a real space point *P* will move outward, which means a decrease in the *x* coordinate of *P*_1_ and an increase of the *x* coordinate in *P*_2_. Note that the temperature-introduced out-of-plane translations of the sensor and lens were clearly observed during a real test^22^. As a result, the disparity (i.e., their difference in *x* coordinate) of these two projection points in *x* direction will increase due to the camera translation. During the calculation, the calibrated camera intrinsic and extrinsic parameters were used to build a world coordinate system, as shown in Fig. 2(b). As all the parameters are fixed for all the images recorded in one test, the temperature-dependent disparities undoubtedly will result in temperature-dependent 3D coordinates, and these varying 3D coordinates further lead to temperature-dependent displacements and strains.

### Image coordinate drifts due to camera self-heating

#### Image coordinate drift in a single camera due to camera self-heating

**Figure 3:**
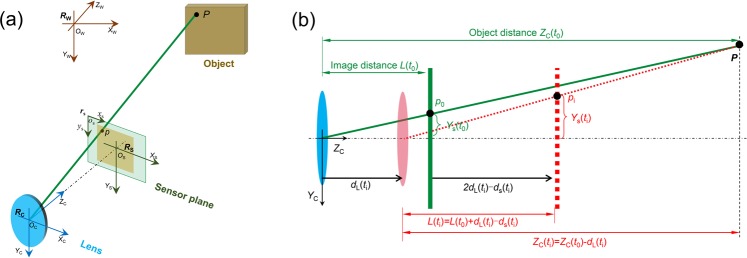
(**a**)A simplified pinhole model and the corresponding coordinate systems, (**b**) the imaging model of the camera before and after camera heating in *Y*_C_*O*_C_*Z*_C_ plane.

To find the connections between the stereo-DIC measurement and the temperature variations, the image coordinate drift (or image distortion) in each camera due to camera self-heating should be modeled. Figure 3(a) shows a simplified pinhole model, which also neglect the lens distortion and skew of the camera sensor. Based on the ideal pinhole model, the relationship between a 3D space point and its projection in sensor plane can be built through three coordinate transformations: (1) from the world coordinate system (*R*_W_) to the camera coordinate system (*R*_C_), (2) from the camera coordinate system (*R*_C_) to sensor plane coordinate system (*R*_S_) and (3) from the sensor plane coordinate system (*R*_S_) to the image plane coordinate system (*r*_s_). By combining these three transformations, the final form of the transformation between the coordinates (*X*_W_, *Y*_W_, *Z*_W_) of a space point *P* in the world coordinate system (*R*_W_) and its coordinates (*x*_s_, *y*_s_) in image plane coordinate system (*r*_s_) can be written as^29^,

3 $$[\begin{array}{c}{x}_{s}\\ {y}_{s}\\ 1\end{array}]=\frac{1}{{Z}_{C}}[\begin{array}{ccc}\frac{L}{{d}_{x}} & 0 & {C}_{x}\\ 0 & \frac{L}{{d}_{y}} & {C}_{y}\\ 0 & 0 & 1\end{array}]([\begin{array}{ccc}{R}_{11} & {R}_{12} & {R}_{13}\\ {R}_{21} & {R}_{22} & {R}_{23}\\ {R}_{31} & {R}_{32} & {R}_{33}\end{array}][\begin{array}{c}{X}_{W}\\ {Y}_{W}\\ {Z}_{W}\end{array}]+[\begin{array}{c}{T}_{x}\\ {T}_{y}\\ {T}_{z}\end{array}])$$

where *Z*_C_ is the *Z*-directional coordinate of *P* in the camera coordinate system (*R*_C_), *L* is the distance between the sensor plane and the optical center (which is usually denoted as *f*, i.e., focal length, units: mm), *d*_*x*_ and *d*_*y*_ are scale factors (units: mm/pixel) relating pixels to distance, *C*_*x*_ and *C*_*y*_ are coordinates (units: pixel) of principal point in image plane coordinate system (*r*_s_), ***R*** = (*R*_11_ *R*_12_ *R*_13_; *R*_21_ *R*_22_ *R*_23_; *R*_31_ *R*_32_ *R*_33_) and ***T*** = (*T*_*x*_ *T*_*y*_ *T*_*z*_)^T^ are the rotation matrix and translation vector between the world coordinate system (*R*_W_) and the camera coordinate system (*R*_C_). Note that the rotation matrix ***R*** can be expressed as^19^,

4 $${\bf{R}}=[\begin{array}{ccc}{R}_{11} & {R}_{12} & {R}_{13}\\ {R}_{21} & {R}_{22} & {R}_{23}\\ {R}_{31} & {R}_{32} & {R}_{33}\end{array}]=[\begin{array}{ccc}\cos \,\beta \,\cos \,\gamma  & \sin \,\alpha \,\sin \,\beta \,\cos \,\gamma -\,\cos \,\alpha \,\sin \,\gamma  & \cos \,\alpha \,\sin \,\beta \,\cos \,\gamma +\,\sin \,\alpha \,\sin \,\gamma \\ \cos \,\beta \,\sin \,\gamma  & \sin \,\alpha \,\sin \,\beta \,\sin \,\gamma +\,\cos \,\alpha \,\cos \,\gamma  & \cos \,\alpha \,\sin \,\beta \,\sin \,\gamma -\,\sin \,\alpha \,\cos \,\gamma \\ -\,\sin \,\beta  & \sin \,\alpha \,\cos \,\beta  & \cos \,\alpha \,\cos \,\beta \end{array}]$$

where *α*, *β* and *γ* are the Euler angles rotating around *X*_W_, *Y*_W_ and *Z*_W_ axes. It should be emphasized that the sensor plane coordinate system (*X*_S_*O*_S_*Y*_S_) is assumed to be parallel and concentric to the *X*_C_*O*_C_*Y*_C_ plane of the camera coordinate system in Eq. (3) for an ideal camera model.

Usually, all the parameters (including the object distance *Z*_C_, the image distance *L*, and the rotation matrix ***R*** and translation vector ***T*** between the world coordinate system and the camera coordinate system) in Eq. (3) should be constant during an ideal test. However, in a real test, these parameters will be changed by the translations and/or rotations of the camera sensor and imaging lens due to the camera self-heating. As a result, these changes will lead to a drift in the image coordinates (*x*_s_, *y*_s_) of projection point *p*, and further cause errors in the stereo-DIC measurement according to the analysis in the last section. To quantitatively analyze the displacement and strain errors in stereo-DIC induced by the camera self-heating, the drift of the projection point in the two cameras should be modeled. As evidenced in refs ^22,24^, the translations of camera lens and sensor plane along the optical axis direction (i.e., *Z*_*C*_ direction) are the dominant factors caused by the camera self-heating and these observations will help to simplify the analysis of the thermal errors in stereo-DIC. Based on these experimental investigations, here we assume the original image distance and object distance are *L*(*t*_0_) and *Z*_C_(*t*_0_) at initial time (*t* = *t*_0_), the thermal-induced out-of-plane translations of the sensor plane and the lens are *d*_S_(*t*_i_) and *d*_L_(*t*_i_) during the test (*t* = *t*_i_), as shown in Fig. 3(b). Note that the values of *d*_S_(*t*_i_) and *d*_L_(*t*_i_) may differ from one camera to another due to the small differences in camera structures. Then the image distance *L*(*t*_i_) and object distance *Z*_C_(*t*_i_) of the changed imaging model can be determined through the geometric relationships. Also, the translation vector ***T*** between the world coordinate system and the camera coordinate system will be changed by the out-of-plane translation of the imaging lens, while the rotation matrix ***R*** is assumed to be constant during the test. With these changed parameters, the locations of the projection point *p* on the sensor plane can be expressed using the imaging model as,

5 $$[\begin{array}{c}{x}_{s}({t}_{i})\\ {y}_{s}({t}_{i})\\ 1\end{array}]=\frac{1}{{Z}_{C}({t}_{0})-{d}_{L}({t}_{i})}[\begin{array}{ccc}\frac{L({t}_{0})+{d}_{L}({t}_{i})-{d}_{S}({t}_{i})}{{d}_{x}} & 0 & {C}_{x}\\ 0 & \frac{L({t}_{0})+{d}_{L}({t}_{i})-{d}_{S}({t}_{i})}{{d}_{y}} & {C}_{y}\\ 0 & 0 & 1\end{array}]([\begin{array}{ccc}{R}_{11} & {R}_{12} & {R}_{13}\\ {R}_{21} & {R}_{22} & {R}_{23}\\ {R}_{31} & {R}_{32} & {R}_{33}\end{array}][\begin{array}{c}{X}_{W}\\ {Y}_{W}\\ {Z}_{W}\end{array}]+[\begin{array}{c}{T}_{x}\\ {T}_{y}\\ {T}_{z}-{d}_{L}({t}_{i})\end{array}])$$

#### Image coordinate drifts in a stereo-DIC system due to camera self-heating

**Figure 4:**
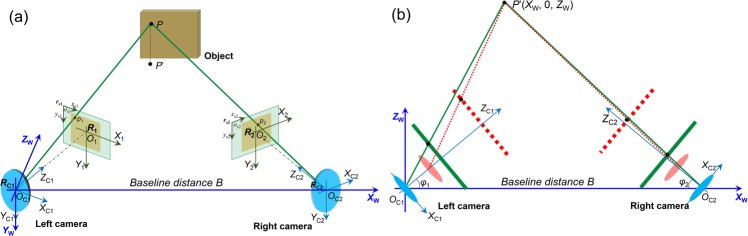
(**a**) Schematic diagram of the imaging model of a stereo-DIC system and (**b**) the changes of the image model in *X*_W_*O*_W_*Z*_W_ plane due to camera self-heating.

Left camera: Based on the analysis in last section, we can associate the image coordinate drifts of the two projection points in a stereo-DIC system with a space point on the object surface. Figure 4 shows the schematic diagram of the imaging model of a stereo-DIC system and the changes of the image model in *X*_W_*O*_W_*Z*_W_ plane due to the camera self-heating. Same as the previous analysis, the world coordinate system is also moved to the optical center of left camera. As shown in Fig. 4, the *Y* axis (*Y*_W_) and original point (*O*_W_) of the world coordinate system coincide to those of the left camera coordinate system (*O*_C1_-*X*_C1_*Y*_C1_*Z*_C1_), while the angle between the *Z* axes of these two coordinate systems can be determined as *β*_L_ = −(90° − *φ*_1_). As a result, the rotation matrix ***R***^***L***^ between the world coordinate system and the left camera coordinate system can be determined by Eq. (4), and the translation vector ***T***^***L***^ is determined as (0 0 0)^T^. Then the mathematical relationship between the global coordinates (*X*_W_, *Y*_W_, *Z*_W_) of a space point and its projection point in left camera sensor (*x*_1_, *y*_1_) at initial time (*t* = *t*_0_) can be expressed as,

6 $$[\begin{array}{c}{x}_{1}({t}_{0})\\ {y}_{1}({t}_{0})\\ 1\end{array}]=\frac{1}{{Z}_{C1}({t}_{0})}[\begin{array}{ccc}\frac{{L}_{1}({t}_{0})}{{d}_{x1}} & 0 & {C}_{x1}\\ 0 & \frac{{L}_{1}({t}_{0})}{{d}_{y1}} & {C}_{y1}\\ 0 & 0 & 1\end{array}]([\begin{array}{ccc}\sin \,{\phi }_{1} & 0 & -\,\cos \,{\phi }_{1}\\ 0 & 1 & 0\\ \cos \,{\phi }_{1} & 0 & \sin \,{\phi }_{1}\end{array}][\begin{array}{c}{X}_{W}\\ {Y}_{W}\\ {Z}_{W}\end{array}]+[\begin{array}{c}0\\ 0\\ 0\end{array}])$$

where *L*_1_(*t*_0_) is the image distance of the left camera at initial time (i.e., the focal length), *Z*_C1_(*t*_0_) is the object distance of the left camera and can be estimated as $${Z}_{C1}({t}_{0})=\sqrt{{X}_{W}^{2}+{Z}_{W}^{2}}\,\cos (\arctan \frac{{Z}_{W}}{{X}_{W}}-{\phi }_{1})={X}_{W}$$$$\cos \,{\phi }_{1}+{Z}_{W}\,\sin \,{\phi }_{1}$$ through the geometric relationships in triangle *P*’*O*_C1_*O*_C2_.

When the camera is working, we assume the out-of-plane translations of the sensor and the lens of the left camera at the time of *t*_i_ are denoted as *d*_S1_(*t*_i_) and *d*_L1_(*t*_i_), respectively. The rotation matrix ***R***^***L***^ between the world coordinate system and the left camera coordinate system should be kept stable as the relative rotation is neglected, while the translation vector ***T***^***L***^ will be changed to $${T}^{L}={(\begin{array}{ccc}0 & 0 & -{d}_{L1}({t}_{i})\end{array})}^{T}$$ in the left camera coordinate system. Then the mathematical relationship between the global coordinates (*X*_W_, *Y*_W_, *Z*_W_) of a space point and its projection point in left camera sensor (*x*_1_, *y*_1_) during the test (*t* = *t*_i_) can be written as,

7 $$[\begin{array}{c}{x}_{1}({t}_{i})\\ {y}_{1}({t}_{i})\\ 1\end{array}]=\frac{1}{{Z}_{C1}({t}_{0})-{d}_{L1}({t}_{i})}[\begin{array}{ccc}\frac{{L}_{1}({t}_{0})+{d}_{L1}({t}_{i})-{d}_{S1}({t}_{i})}{{d}_{x1}} & 0 & {C}_{x1}\\ 0 & \frac{{L}_{1}({t}_{0})+{d}_{L1}({t}_{i})-{d}_{S1}({t}_{i})}{{d}_{y1}} & {C}_{y1}\\ 0 & 0 & 1\end{array}]([\begin{array}{ccc}\sin \,{\phi }_{1} & 0 & -\,\cos \,{\phi }_{1}\\ 0 & 1 & 0\\ \cos \,{\phi }_{1} & 0 & \sin \,{\phi }_{1}\end{array}][\begin{array}{c}{X}_{W}\\ {Y}_{W}\\ {Z}_{W}\end{array}]+[\begin{array}{c}0\\ 0\\ -{d}_{L1}({t}_{i})\end{array}])$$

Right camera: For the right camera, the *Y*-axis (*Y*_W_) of the world coordinate system coincide to that (*Y*_C2_) of the right camera coordinate system (*O*_C2_-*X*_C2_*Y*_C2_*Z*_C2_), while the angle between the *Z* axes of these two coordinate systems is estimated as *β*_R_ = (90° − *φ*_2_). As a result, the rotation matrix ***R***^***R***^ and the translation vector ***T***^***R***^ between the world coordinate system and the right camera coordinate system can be calculated. Then the mathematical relationship between the global coordinates (*X*_W_, *Y*_W_, *Z*_W_) of the space point and its projection point in right camera sensor (*x*_2_, *y*_2_) at initial time (*t* = *t*_0_) can be expressed as,

8 $$[\begin{array}{c}{x}_{2}({t}_{0})\\ {y}_{2}({t}_{0})\\ 1\end{array}]=\frac{1}{{Z}_{C2}({t}_{0})}[\begin{array}{ccc}\frac{{L}_{2}({t}_{0})}{{d}_{x2}} & 0 & {C}_{x2}\\ 0 & \frac{{L}_{2}({t}_{0})}{{d}_{y2}} & {C}_{y2}\\ 0 & 0 & 1\end{array}]([\begin{array}{ccc}\sin \,{\phi }_{2} & 0 & \cos \,{\phi }_{2}\\ 0 & 1 & 0\\ -\,\cos \,{\phi }_{2} & 0 & \sin \,{\phi }_{2}\end{array}][\begin{array}{c}{X}_{W}\\ {Y}_{W}\\ {Z}_{W}\end{array}]+[\begin{array}{c}-B\,\sin \,{\phi }_{2}\\ 0\\ B\,\cos \,{\phi }_{2}\end{array}])$$

where *L*_2_(*t*_0_) is the image distance of the right camera at the initial time (i.e., the focal length), *Z*_C2_(*t*_0_) is the object distance of the right camera and estimated as ${Z}_{C2}({t}_{0})=\sqrt{{(B-{X}_{W})}^{2}+{Z}_{W}^{2}}\,\cos (\arctan \frac{{Z}_{W}}{B-{X}_{W}}-{\phi }_{2})=(B-{X}_{W})$ $\cos \,{\phi }_{2}+{Z}_{W}\,\sin \,{\phi }_{2}$.

Similarly, we assume the out-of-plane translations of the sensor and the lens of the right camera at the time of *t*_i_ are *d*_S2_(*t*_i_) and *d*_L2_(*t*_i_) when the camera is working, respectively. The rotation matrix ***R***^***R***^ between the world coordinate system and the right camera coordinate system is also assumed not changed, while the translation vector ***T***^***R***^ will be changed to $${T}^{R}={(\begin{array}{ccc}-B\sin {\phi }_{2} & 0 & B\cos {\phi }_{2}-{d}_{L2}({t}_{i})\end{array})}^{T}$$ in the right camera coordinate system. Then the mathematical relationship between the global coordinates (*X*_W_, *Y*_W_, *Z*_W_) of a space point and its projection point in right camera sensor (*x*_2_, *y*_2_) during the test (*t* = *t*_i_) can be written as,

9 $$[\begin{array}{c}{x}_{2}({t}_{i})\\ {y}_{2}({t}_{i})\\ 1\end{array}]=\frac{1}{{Z}_{C2}({t}_{0})-{d}_{L2}({t}_{i})}[\begin{array}{ccc}\frac{{L}_{2}({t}_{0})+{d}_{L2}({t}_{i})-{d}_{S2}({t}_{i})}{{d}_{x2}} & 0 & {C}_{x2}\\ 0 & \frac{{L}_{2}({t}_{0})+{d}_{L2}({t}_{i})-{d}_{S2}({t}_{i})}{{d}_{y2}} & {C}_{y2}\\ 0 & 0 & 1\end{array}]([\begin{array}{ccc}\sin \,{\phi }_{2} & 0 & \cos \,{\phi }_{2}\\ 0 & 1 & 0\\ -\,\cos \,{\phi }_{2} & 0 & \sin \,{\phi }_{2}\end{array}][\begin{array}{c}{X}_{W}\\ {Y}_{W}\\ {Z}_{W}\end{array}]+[\begin{array}{c}-B\,\sin \,{\phi }_{2}\\ 0\\ B\,\cos \,{\phi }_{2}-{d}_{L2}({t}_{i})\end{array}])$$

### Thermal errors in 3D coordinate, displacement and strain measurements

With above equations, the image coordinates of the two projections (*p*_1_, *p*_2_) on the two camera sensors before and during the camera working process can be estimated provided that the translations of the camera lenses and sensors are measured or pre-estimated. By substituting these temperature-dependent image coordinates into Eq. (1), the reconstructed 3D coordinates of the space point *P* can be retrieved, and will also present a temperature-dependent trend according to the previous analysis. Then, based on these reconstructed 3D coordinates at different times, the displacement and strain errors caused by the camera self-heating can be evaluated.

#### Errors in 3D coordinate reconstruction

For a specific space point *P* (*X*_W_, *Y*_W_, *Z*_W_, in the world coordinate system defined at the optical center of the left camera), the drifted image coordinates of the two projection points can be estimated through Eqs (7) and (9). By substituting these temperature-dependent image coordinates into Eq. (1), the reconstructed 3D coordinates of the space point *P* at the time of *t*_i_ can be written as,

10 $$\begin{array}{c}{X}_{W}({t}_{i})=\frac{B}{\cot \,{\theta }_{1}({t}_{i})+\,\cot \,{\theta }_{2}({t}_{i})}\,\cot \,{\theta }_{1}({t}_{i})\\ {Y}_{W}({t}_{i})=\frac{B}{\cot \,{\theta }_{1}({t}_{i})+\,\cot \,{\theta }_{2}({t}_{i})}\frac{\tan \,{\alpha }_{1}({t}_{i})}{\sin \,{\theta }_{1}({t}_{i})}\\ {Z}_{W}({t}_{i})=\frac{B}{\cot \,{\theta }_{1}({t}_{i})+\,\cot \,{\theta }_{2}({t}_{i})}\end{array}$$

where the angles can be represented as,

11 $$\begin{array}{c}{\theta }_{1}({t}_{i})=\arctan \frac{{x}_{1}({t}_{i})-{C}_{x1}}{{f}_{1}/{d}_{x1}}+{\phi }_{1}\\ {\theta }_{2}({t}_{i})=\arctan \frac{{x}_{2}({t}_{i})-{C}_{x2}}{{f}_{2}/{d}_{x2}}+{\phi }_{2}\\ {\alpha }_{1}({t}_{i})=\arctan (\frac{{y}_{1}({t}_{i})-{C}_{y1}}{{f}_{1}/{d}_{y1}}\,\cos (\arctan \frac{{x}_{1}({t}_{i})-{C}_{x1}}{{f}_{1}/{d}_{x1}}))\end{array}$$

Note that *f*_1_ and *f*_2_ are the focal lengths of the left and right cameras, and are equal to *L*_1_(*t*_0_) and *L*_2_(*t*_0_), *B* is the baseline distance, *φ*_1_ and *φ*_2_ are the angles between the optical axes of the left and right cameras and the baseline.

#### Errors in displacement and strain measurements

By subtracting the reconstructed 3D coordinates at the time of *t*_i_ from those at the time of *t*_0_, the 3D displacement errors of the space point *P* can be derived as,

12 $$\begin{array}{c}U({t}_{i})={X}_{W}({t}_{i})-{X}_{W}({t}_{0})=\frac{B}{\cot \,{\theta }_{1}({t}_{i})+\,\cot \,{\theta }_{2}({t}_{i})}\,\cot \,{\theta }_{1}({t}_{i})-{X}_{W}({t}_{0})=B[\frac{\cot \,{\theta }_{1}({t}_{i})}{\cot \,{\theta }_{1}({t}_{i})+\,\cot \,{\theta }_{2}({t}_{i})}-\frac{\cot \,{\theta }_{1}({t}_{0})}{\cot \,{\theta }_{1}({t}_{0})+\,\cot \,{\theta }_{2}({t}_{0})}]\\ V({t}_{i})={Y}_{W}({t}_{i})-{Y}_{W}({t}_{0})=\frac{B}{\cot \,{\theta }_{1}({t}_{i})+\,\cot \,{\theta }_{2}({t}_{i})}\frac{\tan \,{\alpha }_{1}({t}_{i})}{\sin \,{\theta }_{1}({t}_{i})}-{Y}_{W}({t}_{0})=B[\frac{\tan \,{\alpha }_{1}({t}_{i})/\sin \,{\theta }_{1}({t}_{i})}{\cot \,{\theta }_{1}({t}_{i})+\,\cot \,{\theta }_{2}({t}_{i})}-\frac{\tan \,{\alpha }_{1}({t}_{0})/\sin \,{\theta }_{1}({t}_{0})}{\cot \,{\theta }_{1}({t}_{0})+\,\cot \,{\theta }_{2}({t}_{0})}]\\ W({t}_{i})={Z}_{W}({t}_{i})-{Z}_{W}({t}_{0})=\frac{B}{\cot \,{\theta }_{1}({t}_{i})+\,\cot \,{\theta }_{2}({t}_{i})}-{Z}_{W}({t}_{0})=B[\frac{1}{\cot \,{\theta }_{1}({t}_{i})+\,\cot \,{\theta }_{2}({t}_{i})}-\frac{1}{\cot \,{\theta }_{1}({t}_{0})+\,\cot \,{\theta }_{2}({t}_{0})}]\end{array}$$

After repeating this procedure for all the calculation points evenly spaced in the pre-defined region of interest, the 3D displacement error fields at different times can be determined. By differentiating the displacement fields using a pointwise least square strain estimation approach, the full-field strain error fields at each configuration can be calculated.

## Simulation of The Errors in Stereo-DIC Due to Camera Self-Heating

### Effect of camera self-heating on the 3D coordinate, displacement and strain measurements

To get a first knowledge of the error distributions and trends in stereo-DIC due to camera self-heating, a simulation test is first conducted. According to the aforementioned analysis, the camera parameters to establish the world coordinate system, the out-of-plane translations of the sensor and lens due to camera self-heating and the global coordinates of the test object in the world coordinates are the required inputs for error estimations. To simplify the simulation process, the camera parameters refer to the system configuration of a real stereo-DIC system^25^, and the out-of-plane translations of the camera sensor and camera lens per Celsius are taken from the experimental observations^22^. The test object is a planar surface with a physical dimension of 80 mm × 80 mm, and placed in front of the system with a working distance of 730 mm. All these inputs for the simulation are summarized in Table 1. With these inputs, the errors in stereo-DIC caused by the camera self-heating can be estimated through the above equations. Note that, to simulate the matching uncertainties in stereo-matching, zero-mean random noise with a standard deviation of 0.001 pixel was added to the image coordinates of the two projection points in left and right camera sensors.

**Table 1:** The assumed camera parameters, out-of-plane translations of the sensor and lens and global coordinates of the test object for the error estimation in stereo-DIC.

	Inputs	Left camera		Right camera	
1	Camera parameters	*f*_1_(mm)	50	*f*_2_(mm)	50
		*C*_*x*1_(pixel)	1224	*C*_*x*2_(pixel)	1224
		*C*_*y*1_(pixel)	1024	*C*_*y*2_(pixel)	1024
		*d*_*x*1_(mm/pixel)	3.45 × 10^−3^	*d*_*x*2_(mm/pixel)	3.45 × 10^−3^
		*d*_*y*1_(mm/pixel)	3.45 × 10^−3^	*d*_*y*2_(mm/pixel)	3.45 × 10^−3^
		*φ*_1_(°)	76	*φ*_2_(°)	76
		Baseline distance *B* = 330 mm			
2	Translation of sensor	*d*_*S*1_(*t*_i_) = *k*_S1_ × *T*(*t*_i_), *k*_S1_ = 3.1μm/°C		*d*_*S*2_(*t*_i_) = *k*_S2_ × *T*(*t*_i_), *k*_S2_ = 3.1μm/°C	
	Translation of lens	*d*_*L*1_(*t*_i_) = *k*_L1_ × *T*(*t*_i_), *k*_L1_ = 4.4μm/°C		*d*_*L*2_(*t*_i_) = *k*_L2_ × *T*(*t*_i_), *k*_L2_ = 4.4μm/°C	
		*T*(*t*_i_) is the temperature change curve taken from a real test			
3	Global coordinates	Planar surface (*X*_W_ = 125:1:205, *Y*_W_ = −40:1:40, *Z*_W_ = 730, units: mm)			

**Figure 5:**
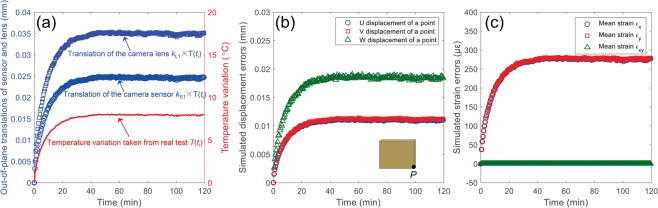
(**a**) A typical temperature variation curve in the imaging camera after switched on and the estimated out-of-plane translations of the camera sensor and imaging lens, (**b**) 3D displacement errors of a physical point *P*(205, 40, 730) on the specimen surface and (**c**) the measured mean in-plane strain errors as a function of time.

Figure 5(a) shows a typical temperature variation curve in the imaging camera after switched on and the estimated out-of-plane translations of the camera sensor and imaging lens based on the existed investigation^22^. With these temperature-dependent out-of-plane translations and the camera parameters, the temperature-dependent image coordinates of the two projections of the physical points on the object surface can be determined through Eqs (7) and (9). Based on the temperature-dependent image coordinates of the two projections and the assumed constant camera parameters, the 3D coordinates of the physical points can be reconstructed. By subtracting the reconstructed 3D coordinates at different time from the 3D coordinates reconstructed at the initial time (i.e., as indicated in Eq. (12)), the full-field 3D displacement errors of the test points were determined. Figure 5(b) shows the 3D displacement errors of a point *P*(205, 40, 730) in all the test points as a function of time. Clearly, the *U*, *V* and *W* displacement errors all present a similar trend with the temperature and *W* displacement error is larger than those of *U* and *V*. Then, the corresponding strain errors fields can be evaluated by differentiating the displacement fields and the mean in-plane strains are plotted in Fig. 5(c). It is observed that normal strain-time curves show a strong positive correlation with the temperature variations and the values of ε_*x*_ and ε_*y*_ are almost identical, while the in-plane shear strains are always close to zero. These observations are in good accordance with the experimental results in the previous studies^25^.

**Figure 6:**
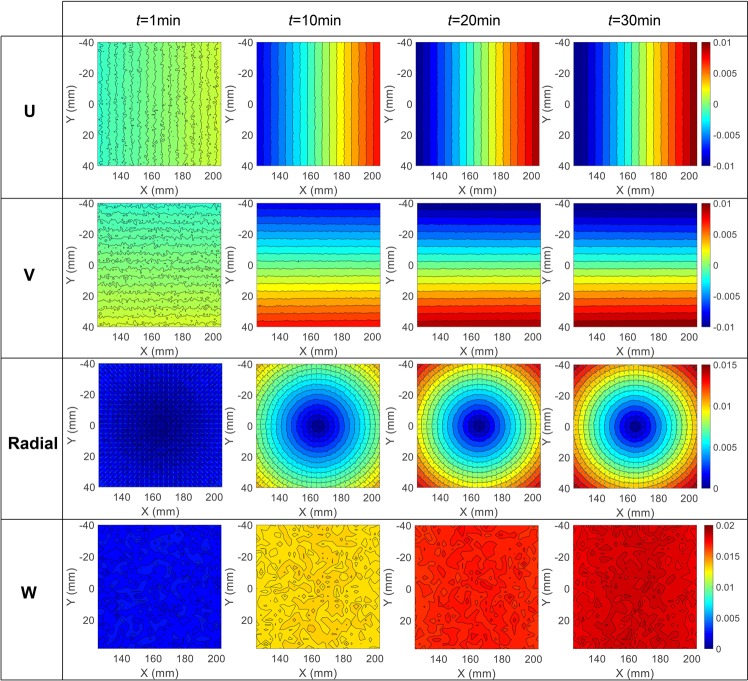
Simulated full-field *U*, *V*, radial and *W* displacement error fields at the time of 1st min, 10th min, 20th min and 30th min based on the assumed camera parameters and camera components translations.

To intuitively present the effect of camera self-heating on stereo-DIC measurement, the full-field *U*, *V*, radial $(\sqrt{{U}^{2}+{V}^{2}})$ and *W* displacement error fields at the times of 1st min, 10th min, 20th min and 30th min were given in Fig. 6. As shown in these figures, the *U* and *V* displacement error fields are evenly distributed along *X* and *Y* directions, indicating a virtual tensile strain in both directions. The dilatational deformations are more evident from the concentric radial displacement error fields. Ideally, the mean *U* and *V* displacements should be close to zeros if the center of the ROI exactly located at the perpendicular bisector of the baseline in *XOY* plane. Once the ROI deviate from the perpendicular bisector, the mean *U* and *V* displacements will not be zero and will change with the camera temperature. Also, it is not difficult to conclude from these displacement fields that the tensile strains in *X* and *Y* directions are almost equal, and the in-plane shear strain is negligible. In addition, it is interestingly to find that the *W* displacement errors at each time vary a little across the whole specimen surface, but will distinctly increase with temperature.

### Effect of camera parameters on the thermal errors in stereo-DIC

As the above results only focus on the errors in a specific stereo-DIC system due to the varying temperature, the effects of the camera parameters on these errors are still not clear. To address this problem, we simulated the errors in stereo-DIC systems with varying camera parameters (i.e., focal length *f*, baseline distance *B*, working distance *D* and included angle *φ*) when the camera sensor and camera lens undergo certain out-of-plane translations at a specific time (e.g., at 60th min). By comparing the measured displacement and strain errors in the stereo-DIC systems with different configurations, the effect of camera parameters on the thermal errors is revealed. It should be noted that two assumptions were made during the simulation. First, the matching accuracy is assumed to be constant and not affected by the other parameters (e.g., shape function, subset size and speckle size, etc.). Then, the sizes of the sensor planes are assumed to be large enough to accommodate the projections of the test object as the projection may go beyond the sensor boundary in some cases. With these two assumptions and the parameters in Table 1, we calculated the thermal displacement and strain errors for the stereo-DIC with different focal length, baseline distance, working distance and included angle, respectively.

**Figure 7:**
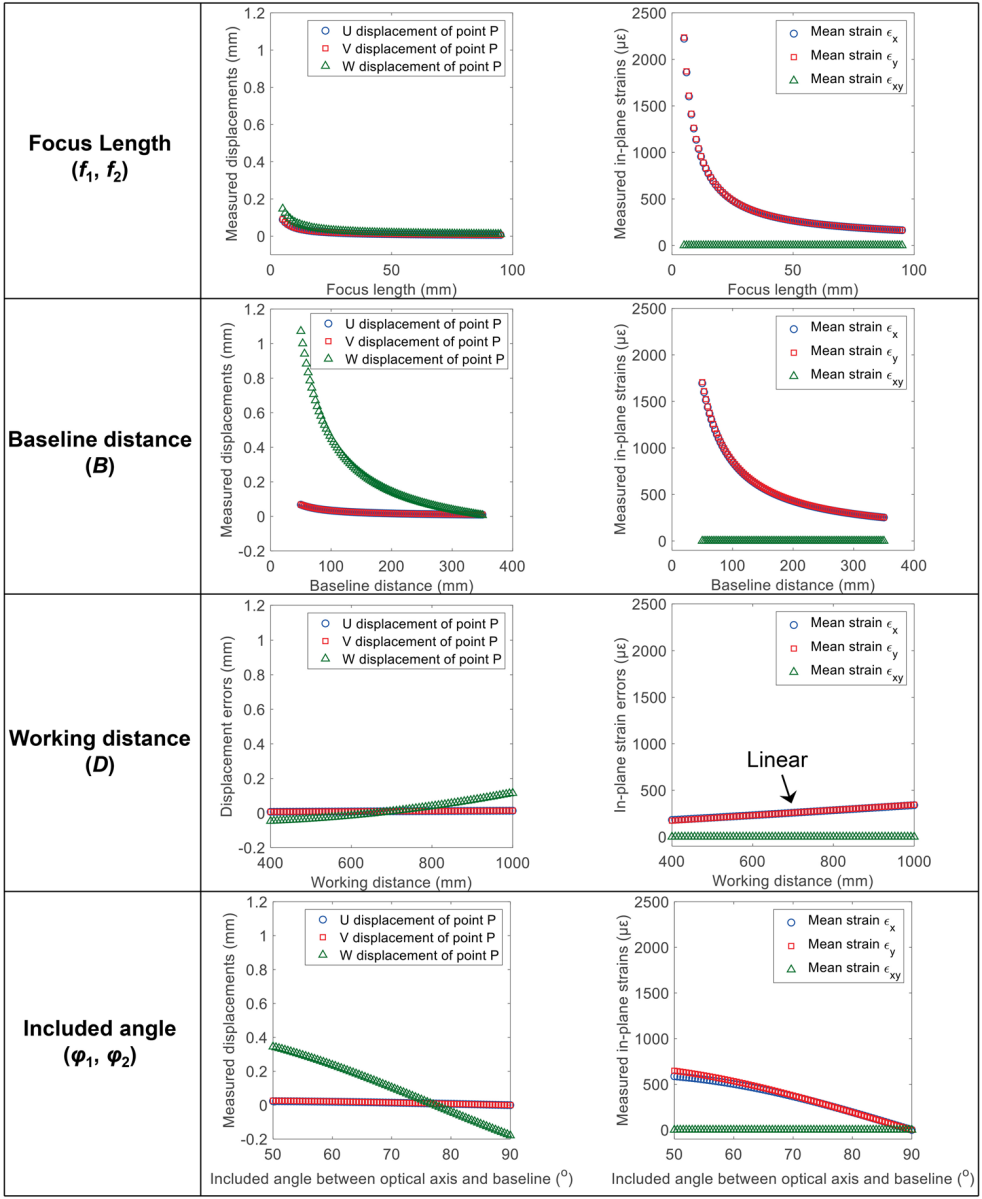
Simulated 3D displacement error of the point *P* and in-plane strain errors as a function of the camera parameters, including focal length, baseline distance, working distance and included angle. The translations of the camera lenses and sensors are constant and taken from the data in Fig. 5(a) at the time of 60th min.

Figure 7 shows the simulated 3D displacement error of the point *P* and in-plane strain errors as a function of the camera parameters, including focal length, baseline distance, working distance and included angle. Note that, in these simulations, the translations of the camera lenses and sensors are constant and taken from the data in Fig. 5(a) at the time of 60th min. From these figures, it can be concluded that the measured *W* displacement errors are more sensitive to camera parameters than the *U* and *V* displacement errors, especially for the system configuration with small baseline distance and included angle. The 3D displacement errors all decrease with the increase of the focal length and baseline distance, and the *U* and *V* displacement errors are almost unaffected by the working distance and included angle. For the in-plane strain errors, the ε_*x*_ and ε_*y*_ strain errors are almost equal and extremely sensitive to focal length and baseline distance. This can explain why a smartphone-based stereo-DIC system (small baseline distance and short focal length)^26^ is more sensitive to the camera self-heating than the conventional stereo-DIC system^25^. As shown in Fig. 7, the ε_*x*_ and ε_*y*_ strain errors will notably decrease with the increase of focal length and baseline distance, while the in-plane shear strain is always close to zero for different system configuration. Also, it is observed that the ε_*x*_ and ε_*y*_ strain errors are in a positive linear relationship with the working distance, which means a small working distance is preferred for a stereo-DIC system sensitive to the camera self-heating.

## Experimental Verifications

### Experimental details

**Figure 8:**
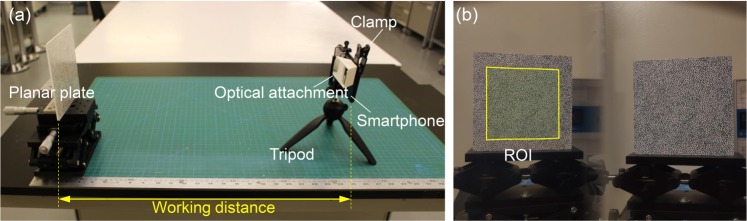
(**a**)Experimental set-up for the validation test and (**b**) a recorded image with defined ROI. The imaging system is a smartphone-based single-camera stereo-DIC system, which has been prone to be effective in 3D deformation measurement and more sensitive to the camera self-heating than a conventional stereo-DIC system.

To verify the correctness of the theoretical analysis of the systematic errors in stereo-DIC due to camera self-heating, a series of self-heating experiments were performed. Figure 8(a) shows the experimental arrangement for the verification tests. Here, we adopted a single-camera stereo-DIC system using a smartphone as the imaging system, which is equivalent to a stereo-DIC system using two same virtual cameras. It is much smaller in focal length and baseline distance than conventional stereo-DIC systems, and thus should be more sensitive to the camera self-heating according to the above analysis. Besides, it should be noted that image model of the smartphone camera is similar with that of a conventional camera, which are both based on the pinhole model. The accuracy of the smartphone-based stereo-DIC system in 3D shape, motion and deformation measurement was successfully verified in our previous study^26^. As shown in Fig. 8(a), the smartphone-based stereo-DIC system is mainly composed of an Android-based smartphone (Mi8, Xiaomi, Inc., China), a homemade optical attachment, and a small tripod (Type 258, Yunteng, Inc., China). The test specimen is a flat plate (150 mm × 150 mm × 5 mm) and placed right in front of the imaging system. Random speckle patterns were decorated onto the specimen surfaces using white spray paints and a black marker pen. The distance between the specimen and the imaging system was first set as 600 mm. During the experiment, the indoor temperature was kept constant, and the planar plate was held static. After that, surface images of the plate were recorded by the smartphone every 30 seconds with the help of an app called Tasker (the smartphone was rooted for emulating touch input). The image recording process lasted for 2 hours and about 240 images were recorded in this test. Note that the imaging system was calibrated before and after self-heating and the temperature variation of the smartphone system during the test was monitored using an infrared thermal camera (SC7900, FLIR). Further, similar experiments were repeated with different working distances, i.e., 300 mm, 450 mm, 750 mm and 900 mm. Finally, to investigate the effect of baseline distance, we conducted an additional self-heating test using the same smartphone and a larger four-mirror device at a working distance of 600 mm.

All the recorded images were then processed to determine the displacement and strain errors due to the camera self-heating using conventional stereo-DIC algorithm. During the calculation, the first image in the image series was adopted as the reference image, and the rest images were regarded as the deformed images. As shown in Fig. 8(b), a rectangular area in the middle of the left image, was selected as the ROI. The displacements were calculated with a subset size of 41 × 41 pixels and a grid step of 10 pixels. Subsequently, a pointwise least squares algorithm using a strain calculation window of 15 × 15 sets of displacement data was employed to estimate the strain fields from the displacement results. As no external loading was applied to the plate, the actual displacements and strains on the plate should be zero and the measured displacements and strains can only be attributed to the errors due to camera self-heating.

## Results

**Figure 9:**
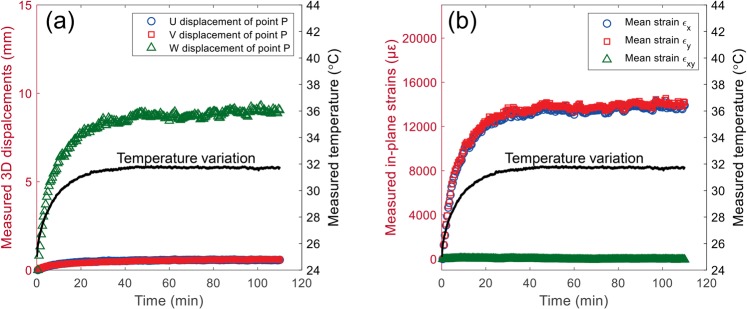
(**a**) 3D displacement errors of a physical point *P* on the specimen surface and (**b**) the measured mean in-plane strain errors as a function of time.

Figure 9(a,b) show the 3D displacement errors of a physical point *P*(65.7, 40.3, 603.8, in world coordinate system) and the measured mean in-plane strain errors on the specimen surface as a function of time, respectively. It should be noted that we compared the camera parameters calibrated before and after self-heating tests, and no noticeable or regular changes were observed. This is because that the variations of the camera parameters due to the nonlinear optimization is not small enough, and may cover up the real changes in the camera parameters. The displacements and strain errors determined from these two different sets of camera parameters are very close. As shown in Fig. 9, the *U*, *V*, *W* displacement and the two normal strain errors all highly correlated with the detected temperature changes on the smartphone back surface. These trends can be separated into two consecutive stages, i.e., the warm-up stage and thermal equilibrium stage. As shown in these two figures, the three displacement components and two normal strains increase rapidly with the temperature in the first stage and then stabilize at certain values in the thermal equilibrium stage. These observations are consistent with the simulated displacement and strain errors and the results in existing researches^25^. The only difference is that the amplitudes of the displacement and strain errors in this smartphone-based stereo-DIC system are much larger than those in a conventional stereo-DIC system, which can be well explained by the above simulation results. Compared with the conventional stereo-DIC system in the simulation section (*f*_1_ = *f*_2_ = 50 mm, *B* = 330 mm), the focal length and the baseline distance of the smartphone-based stereo-DIC system (*f*_1_ = *f*_2_ = 5 mm, *B* = 50 mm) are much smaller. According to the simulation results in Fig. 7, the displacement and strain errors in this smartphone-based stereo-DIC system are unavoidably much larger.

**Figure 10:**
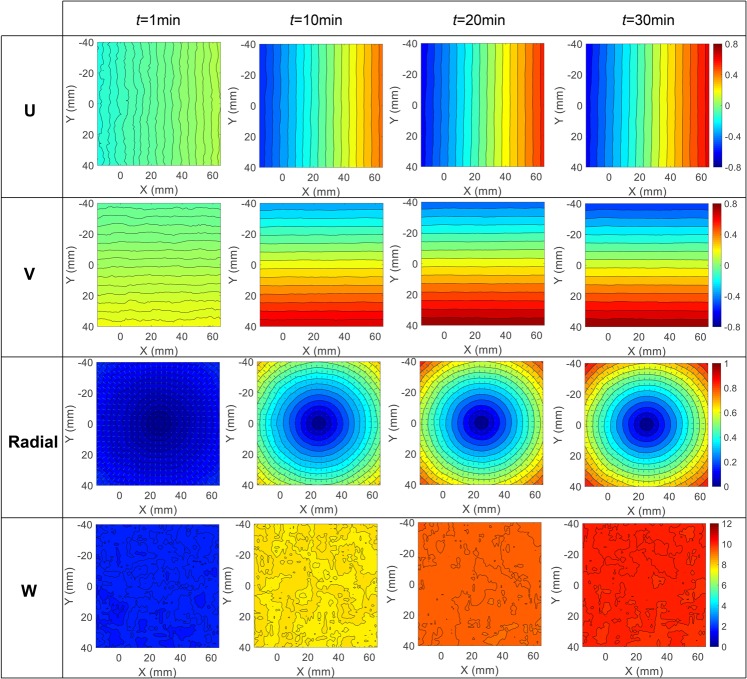
Measured full-field *U*, *V*, radial and *W* displacement error fields at the time of 1st min, 10th min, 20th min, and 30th min during the self-heating test with a working distance of 600 mm.

Figure 10 shows the measured full-field *U*, *V*, radial and *W* displacement error fields at the time of 1st min, 10th min, 20th min and 30th min during the self-heating test with a working distance of about 600 mm. Same with the simulated displacement errors shown in Fig. 6, the measured *U* and *V* displacement error fields in the real test are also evenly distributed along *X* and *Y* directions, respectively. This indicates that the specimen surface undergo a virtual tensile strain in both directions and the virtual strains can be determined from the displacement gradients. The radial displacement. The third row of Fig. 10 shows the resultant displacement fields of the *U* and *V* displacement error fields. The superimposed contour lines in this figure denote the magnitudes of the radial displacement vectors, and the homogeneous thermal expansion is evident. Also, the *W* displacement error fields at 1st min, 10th min, 20th min and 30th min were presented in the last row of this figure. The *W* displacements on the specimen surface are almost equal at each time step, while the amplitudes are much larger than the simulated *W* displacement errors (Fig. 6) of a conventional stereo-DIC system. The reason why the *W* displacement errors in the smartphone-based stereo-DIC system are much larger can be well explained by the simulation analysis.

**Figure 11:**
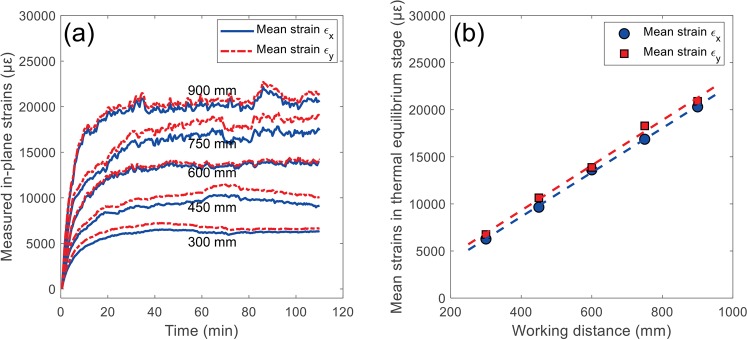
(**a**) Measured in-plane normal strain errors as a function of test time for self-heating tests with five different working distances and (**b**) mean normal strains in thermal equilibrium stage as a function of the working distance.

The thermal errors in the self-heating tests with different working distances were further compared to indicate the effect of working distance. Figure 11(a) shows the measured in-plane normal strain errors as a function of test time for self-heating tests with five different working distances (i.e., 300 mm, 450 mm, 600 mm, 750 mm and 900 mm). It is clearly observed that the thermal strains all present a rapidly increase stage (0~40 min) and an equilibrium stage (40 min~end), and the thermal errors are more significant for the test with longer working distance. The in-plane strain errors in two directions are almost equal and small differences in some tests (e.g., 450 mm and 750 mm) may be attributed to the small translation and/or rotation of the camera. Taking the mean values of the thermal strains in the equilibrium stage as the ordinate values, Fig. 11(b) shows the mean normal strains in thermal equilibrium stage as a function of the working distance. As seen from this figure, the two strain errors almost linearly increase with working distance with the slopes determined as about 23.51 and 23.95 με/mm. These observations are in good accordance with the simulation results in Fig. 7, and the difference in the slopes can be attributed to the differences in camera intrinsic and extrinsic parameters.

**Figure 12:**
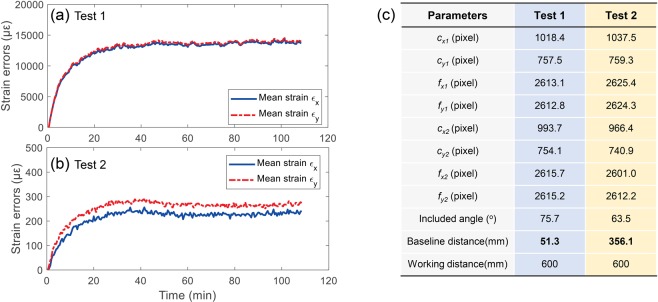
Measured in-plane normal strain errors as a function of test time for the self-heating tests with two different optical attachment: (**a**) original optical attachment shown in Fig. 8, (**b**) a four-mirror device with a larger space between mirrors. The calibrated camera parameters and working distances for these two tests are present in (**c**), where *c*_*x1*_, *c*_*y1*_, *c*_*x2*_ and *c*_*y2*_ are the image coordinates of the principal points for the left and right virtual cameras, *f*_*x1*_, *f*_*y1*_, *f*_*x2*_ and *f*_*y2*_ are the focal lengths in two directions of the two virtual cameras.

Figure 12 shows the measured in-plane normal strain errors as a function of test time for the self-heating tests with two different optical attachments, i.e., the original optical attachment shown in Fig. 8 for test 1 and a four-mirror device with a larger space between mirrors for test 2. Note that the working distances for the two tests were both set as 600 mm. The camera parameters of the systems in these two tests were then calibrated and list in Fig. 12(c). It is clear that the main difference between the two systems is the baseline distances between the two virtual cameras. As shown in Fig. 12(a,b), the measured in-plane normal strain errors all present a similar trend with the temperature variation in the smartphone. However, strain errors in the thermal equilibrium stage of test 1 are significantly larger than those of test 2, which indicates a larger baseline distance will result in smaller thermal errors. This observation is in good accordance with the simulation test.

## Conclusions and Discussions

In this paper, the systematic errors in stereo-DIC due to camera self-heating is investigated theoretically and experimentally. The mechanism of the thermal error generation in stereo-DIC is first explained, and the theoretical errors in 3D coordinate reconstruction and displacement measurement are derived based on a simplified stereovision model. Simulation results explicitly show the effect of camera self-heating on the 3D coordinate, displacement and strain measurements and the effect of camera parameters on the thermal errors in stereo-DIC. Finally, experimental results from a smartphone-based stereo-DIC system successfully verify the correctness of theoretical analyses.

It is worth noting that the thermal errors caused by camera self-heating and/or temperature variation of the environment are unavoidable in all stereovision systems. These errors are usually small enough to be neglected in 3D measurement but should be paid attention to in some cases, e.g., when a high-accuracy measurement is required or using a stereovision system with a small baseline distance and/or a short focal length. To ensure high-accuracy 3D measurement under these circumstances, the test object should be placed near the system as much as possible to reduce the working distance, and solutions that can mitigate these thermal errors are required if the thermal errors are still not small enough to be neglected. Besides, during camera self-heating process, the rotation matrix ***R*** and translation vector ***T*** between the world coordinate system and the camera coordinate system may also be changed by the rigid-body translation and rotation of the camera or even environment vibrations. Also, the position of the principle point may be changed by the camera self-heating. These changes will add additional errors to the measured results. However, compared with the clearly observed out-of-plane translations of the sensor plane and camera lens, the rigid-body translation and rotation of the camera are less obvious and very difficult to measure. Once the rigid-body translation and rotation of the camera are determined, the analytical model in this paper can still be applied.

## Data Availability

The datasets generated during and/or analysed during the current study are available from the corresponding author on reasonable request.

## References

[1] Luo PF, Chao YJ, Sutton MA, Peters WH (1993). Accurate measurement of three-dimensional deformations in deformable and rigid bodies using computer vision. Exp. Mech..

[2] Sutton MA (2007). Three-dimensional digital image correlation to quantify deformation and crack-opening displacement in ductile aluminum under mixed-mode I/III loading. Opt. Eng..

[3] Orteu JJ (2009). 3-D computer vision in experimental mechanics. Opt. Lasers Eng..

[4] Gustafsson A (2018). Linking multiscale deformation to microstructure in cortical bone using *in situ* loading, digital image correlation and synchrotron X-ray scattering. Acta Biomater..

[5] Yu L, Pan B (2018). Experimental study of tensile properties and deformation evolutions of 2D and 2.5D woven SiO_2f_/SiO_2_ composites using single-camera stereo-digital image correlation. Compos. Struct..

[6] Schreier HW, Garcia D, Sutton MA (2004). Advances in light microscope stereo vision. Exp. Mech..

[7] Hu Z, Luo H, Du Y, Lu H (2013). Fluorescent stereo microscopy for 3D surface profilometry and deformation mapping. Opt. Express.

[8] Helfrick MN, Niezrecki C, Avitabile P, Schmidt T (2011). 3D digital image correlation methods for full-field vibration measurement. Mech. Syst. Signal Process..

[9] Kumar P, LeBlanc J, Stargel DS, Shukla A (2012). Effect of plate curvature on blast response of aluminum panels. Int. J. Impact Eng..

[10] Yu L, Pan B (2018). High-speed stereo-digital image correlation using a single color high-speed camera. Appl. Opt..

[11] Pan B, Wu D, Yu L (2012). Optimization of a three-dimensional digital image correlation system for deformation measurements in extreme environments. Appl. Opt..

[12] Berke RB, Sebastian CM, Chona R, Patterson EA, Lambros J (2016). High Temperature Vibratory Response of Hastelloy-X: Stereo-DIC Measurements and Image Decomposition Analysis. Exp. Mech..

[13] Pan B, Yu LP, Zhang QB (2018). Review of single-camera stereo-digital image correlation techniques for full-field 3D shape and deformation measurement. Science China Technological Sciences.

[14] Wong KW, Lew M, Ke Y (1990). Experience with two vision systems. *Close-Range Photogrammetry Meets Machine Vision*. in Int. Soc. Opt. Phot..

[15] Robson S, Clarke TA, Chen J (1993). Suitability of the Pulnix TM6CN CCD camera for photogrammetric measurement. Videometrics II. In Int. Soc. Opt. Phot..

[16] Handel H (2009). Analyzing the Influences of Camera Warm-Up Effects on Image Acquisition. IPSJ Trans. Comput. Vis. Appl..

[17] Podbreznik, P. & Potočnik, B. Influence of Temperature Variations on Calibrated Cameras. *Int*. *J*. *Comput*. *Inf*. *Eng*. **2**(4) (2008).

[18] Podbreznik P, Potočnik B (2012). Assessing the influence of temperature variations on the geometrical properties of a low-cost calibrated camera system by using computer vision procedures. Mach. Vis. Appl..

[19] Yu Q (2014). The effects of temperature variation on videometric measurement and a compensation method. Image Vis. Comput..

[20] Ma S, Pang J, Ma Q (2012). The systematic error in digital image correlation induced by self-heating of a digital camera. Meas. Sci. Technol..

[21] Pan B, Yu L, Wu D (2013). High-accuracy 2D digital image correlation measurements with bilateral telecentric lenses: error analysis and experimental verification. Exp. Mech..

[22] Ma Q, Ma S (2013). Experimental investigation of the systematic error on photomechanic methods induced by camera self-heating. Opt. Express.

[23] Pan B (2018). Thermal error analysis and compensation for digital image/volume correlation. Opt. Lasers Eng..

[24] Ma S, Zhou S, Ma Q (2019). Image distortion of working digital camera induced by environmental temperature and camera self-heating. Opt. Lasers Eng..

[25] Pan B, Shi W, Lubineau G (2015). Effect of camera temperature variations on stereo-digital image correlation measurements. Appl. Opt..

[26] Yu L, Tao R, Lubineau G (2019). Accurate 3D shape, displacement and deformation measurement using a smartphone. Sensors.

[27] Qiong, L., Xiansheng, Q., Shenshun, Y. & Feng, H. Structural parameters optimal design and accuracy analysis for binocular vision measure system. In *IEEE/ASME International Conference on Advanced Intelligent Mechatronics*, *AIM*, 156–161 (2008).

[28] Yang L, Wang B, Zhang R, Zhou H, Wang R (2018). Analysis on Location Accuracy for the Binocular Stereo Vision System. IEEE Photonics J..

[29] Sutton, M. A., Orteu, J. J. & Schreier, H. Image correlation for shape, motion and deformation measurements: Basic concepts, theory and applications. (*Springer Science & Business Media*, 2009).

