# Author Correction: Modeling of systematic errors in stereo-digital image correlation due to camera self-heating

**DOI:** 10.1038/s41598-019-54631-y

**Published:** 2019-11-26

**Authors:** Liping Yu, Gilles Lubineau

**Affiliations:** King Abdullah University of Science and Technology (KAUST), Physical Sciences and Engineering Division (PSE), COHMAS Laboratory, Thuwal, 23955-6900 Saudi Arabia

Correction to: *Scientific Reports* 10.1038/s41598-019-43019-7, published online 25 April 2019

This Article contains a typographical error in Equation 2, where

$${\theta }_{1}=\arctan \frac{{X}_{1}}{{f}_{1}}+{\varphi }_{1}$$

should read:

$${\theta }_{1}=-\,\arctan \frac{{X}_{1}}{{f}_{1}}+{\varphi }_{1}$$

In addition, in Equation 11

$${\theta }_{1}({t}_{i})=arctan\frac{{x}_{1}({t}_{i})-{C}_{x1}}{{f}_{1}/{d}_{x1}}+{\varphi }_{1}$$

should read:

$${\theta }_{1}({t}_{i})=-\,arctan\frac{{x}_{1}({t}_{i})-{C}_{x1}}{{f}_{1}/{d}_{x1}}+{\varphi }_{1}$$

